# Correlates of HIV testing among men in Ghana: Cross-sectional analysis of the 2022 demographic and health survey

**DOI:** 10.1177/09564624251324976

**Published:** 2025-03-01

**Authors:** Sulemana Ansumah Saaka, Roger Antabe, Isaac Luginaah

**Affiliations:** 1 Department of Geography and Environment, Faculty of Social Science, 6221 University of Western Ontario, London, ON, Canada; 2 Department of Health and Society, 33530 University of Toronto Scarborough, Toronto, ON, Canada

**Keywords:** HIV testing, Ghana, Men, sexual behaviour

## Abstract

**Introduction:**

New HIV infections are on the rise in Ghana, with approximately 16,574 new cases reported in 2022 alone. Although HIV prevalence rate is higher among women aged 15–49 years (2.0 [1.7–2.3]) than men aged 15–49 (1.0 [0.8–1.2]) in Ghana, evidence form the country’s 2022 Demographic and health Survey suggest that only 12% of men had ever been tested for HIV once in their lifetime relative to 17% of women, and yet more men (35%) than women (23%) reported having sexual intercourse with persons who were neither their wife nor live-in partners. More so, the Ghana National HIV and AIDS Policy has over the years positively influenced the utilization of HIV testing (HIVT) services among women in Ghana through antenatal care visits. While this policy encourages women to undertake testing with their husbands, most men in the Ghanaian Context rarely accompany their spouse to antenatal care due to several reasons including conflicting work schedules, thus limiting their chances of getting tested. Using a nationally representative dataset, this study adds to the broader literature by exploring the factors associated with HIVT among men.

**Methods:**

Using the 2022 Ghana Demographic and Health Survey (*N* = 7044 males), and employing multiple logistic regression models, this study explored the factors associated with HIVT among men.

**Results:**

Married men (OR = 1.723; *p* < .001), the employed, particularly, those paid in cash only (OR = 2.021; *p* < .001) and those paid both in cash and kind (OR = 1.823; *p* < .001), those who had knowledge of HIV test kits (OR = 1.708; *p* < .001), aware and approve to use Pre-Exposure Prophylaxis (PrEP) (OR = 1.1280; *p* < .001), as well as those who visited a health facility in the past 6 months (OR = 1.615; *p* < .001), all significantly reported higher odds of testing. Moreover, Educational attainment, age, household wealth, religion, ethnicity, and the region of residence significantly predicted HIVT in the study context.

**Conclusion:**

Socio-demographic, economic, geographic and health-related factors have significant influence on the uptake of HIVT among men in Ghana, thus underscoring the need for tailored interventions that consider diverse contextual factors in HIV prevention and healthcare delivery.

## Introduction

Globally, Human Immunodeficiency Virus/Acquired Immunodeficiency Syndrome (HIV/AIDS) remain a significant public health challenge, and Ghana is no exception. Although Ghana has made considerable progress in the fight against HIV/AIDS, the current HIV prevalence rate is still very alarming, as an estimated 1.7% of the adult population is living with the virus.^1^ In response, the Government of Ghana through the Ghana AIDS Commission (GAC), with support from the World Health Organization and other partners, have emphasized the need for widespread HIV testing (HIVT) and early detection: as these are not only deemed effective strategies for HIV prevention, but also, tend to help improve health outcomes among people living with HIV (PLHIV).^2^ For instance, GAC has over the years made a concerted effort towards universal HIVT by implementing various HIVT strategies, including voluntary counselling and testing (VCT) services, provider-initiated testing and counselling (PITC) in healthcare settings, as well as the integration of HIVT into antenatal care services.^2^ Nonetheless, challenges persist in achieving universal testing coverage; only 63% of the population in the country knew their HIV status as of 2020.^3^ Thus, while these efforts have led to HIVT and treatment services in Ghana being free at all government health hospitals and some selected private health facilities in Ghana,^2^ the country’s most recent Demographic and Health Survey (DHS) report indicates that only 24% of men (relative to 54% of women) have ever been tested for HIV in their lifetime and received the results.^4^ Moreover, although HIV prevalence rate is higher among women aged 15–49 years (2.0 [1.7–2.3]) relative to men aged 15–49 (1.0 [0.8–1.2]) in Ghana,^5^ only 12% of men had been tested for HIV once in their lifetime relative to 17% of women.^4^ Nonetheless, more men (35%) than women (23%) reported having sexual intercourse with persons who were neither their wife nor lived-in-partner.^4^ A trend analysis of knowledge about HIV status and HIVT services utilization in sub-Saharan Africa (SSA) from the year 2000 to 2020, further indicates that testing and knowledge of status is generally lower among men than women.^6^ Specifically, over the 20 years period, knowledge of status among men was reportedly 79% relative to 87% among women, with the largest gap in terms of absolute number of undiagnosed men being those aged 35–49 years.^6^

The observed lower levels of HIVT among men, is to a larger extent, due to less policy attention and fewer opportunities for men testing, relative to women. In the Ghanaian context for instance, the National HIV and AIDS Policy^7^ mandates automatic HIVT for pregnant women utilizing antenatal care (ANC) services as part of efforts to reduce mother-to-child transmission. As a results, in the year 2020, 70% of pregnant women who accessed ANC services, received HIV Testing Services (HTS) with 87% of those testing positive, receiving antiretroviral treatment (ART).^8^ The 2022 Ghana DHS report further indicated that 67% of women who gave birth in the preceding 2 years to the survey, had an HIV test during antenatal care (ANC) and received the test results.^9^ While this policy encourages spousal testing, scholarly evidence suggest that most men in Ghana, particularly those residing in rural areas and from poor socioeconomic backgrounds, rarely accompany their wives to antenatal visits.^10,11^ Available evidence suggest that in instances where some men follow their partners to antenatal care, they stay outside/around the health facility premises rather than being in the consultation room with their partners.^12^ Undoubtedly, the absence of deliberate policy interventions directly targeting men for HIVT, has adverse implications for meeting the UNAIDS 95-95-95 target in Ghana.

Men in Ghana have substantial sexual and reproductive health needs, including the need for prevention and treatment of HIV. Yet these needs remain vastly unfulfilled due to a multiplicity of factors including poor healthcare seeking behaviours among men, unavailability of services focusing on men, or absence of “male friendly” health facilities, expression of wrong masculinity (i.e., notion that having more than one sexual partner makes one a “man”), as well as lack of agreed standards for delivering clinical and preventive services to men.^8^ Notably, global health policies and programmes that focus on disease prevention and care for men are scarce.^13^ Disparities in HIVT rates among different demographic groups may also be due to disparities in socio-economic status, educational attainment, and knowledge about the availability and benefits of HIVT.^14^ Studies further suggest that stigma, discrimination, and misconceptions about HIV/AIDS tend to influence individuals’ decision to seek testing services.^15,16^ More so, community dynamics, social norms,^17^ ethno-religious beliefs,^18^ and local healthcare infrastructure^19^ can significantly influence an individual’s decision to undergo HIVT.

Overall, existing studies in Ghana have largely focused on specific population groups^20–23^ or geographic regions,^24,25^ thus providing limited insight into the broader factors influencing men testing behavior. Furthermore, most studies have concentrated on HIV prevalence rates and clinical outcomes, neglecting the socio-cultural, economic, and contextual factors that shape testing decisions, particularly, among men. Thus, there is a paucity of studies focusing on the determinants of HIVT uptake by men in Ghana^14^ even though men are at higher risk of the disease due to their engagement in multiple sexual relationships. Thus, guided by the Health Belief Model (HBM), and using a nationally representative dataset, this study evaluated the demographic, ethno-religious, socioeconomic, HIV-related knowledge and geographical factors influencing HIVT among men in Ghana.

## Theoretical context

This study draws its theoretical inspiration from the Health Belief Model (HBM), a conceptual framework developed in the early 1950s^26^ to understand “the widespread failure of people to accept disease prevention or screening for the early detection of asymptomatic disease”.^27^ HBM consists of four main dimensions of factors that interact to influence the adoption of health behaviours such as HIVT. These four dimensions include (a) perceived susceptibility to illness; (b) perceived illness severity; (c) perceived benefits of treatment; and (d) perceived barriers to treatment.

Perceived susceptibility to illness denotes the individual’s subjective susceptibility of contracting a health condition such as HIV.^16^ Individuals may differ when it comes to personal feelings about both their vulnerability to a disease or health condition, and their perceived seriousness of it. Thus, perceived illness severity includes an evaluation of both the medical/clinical consequences of the disease (including pain, disability, death) and the possible social consequences (e.g., effects on social relations like marriage, family life, work conditions, etc.,) that may result from contracting HIV/AIDS. Likewise, the perceived benefit of treatment relates to beliefs surrounding effectiveness of health actions such as HIVT, status disclosure, and treatment uptake for reducing the associated threats (e.g., death).^16^ The fourth dimension, perceived barriers to treatment, is a cost-benefit analysis whereby the individual weighs the action’s effectiveness (e.g., good health and sustained life) against possible negative consequences.^27^ A fifth component know as ‘cues to action’ was introduced by Janz and Becker^27^ and several studies^16,28^ have since utilized it. ‘Cues to action’ is the information required to trigger the process of engaging in healthy actions (e.g., contact with a health worker and other demographic factors, such as knowledge of HIV test kits). Cues to action can take various forms, including media messages, personal experiences, advice from healthcare professionals, or observations among others.^16^ Thus, guided by the dimensions of the HBM, this study evaluated the factors associated with men’s behaviour towards HIVT in Ghana given that HIV is more prevalent in the country, yet testing remains low among men.

## Methods

### Data collection

This study draws data from the 2022 Ghana Demographic and Health Survey (DHS), implemented by the Ghana Statistical Service (GSS). Data collection took place from 17 October 2022 to 14 January 2023.^4^ Technical assistance to the Demographic and Health Surveys (DHS) Program was provided by the International Coaching Federation (ICF). Ethical clearance for the survey was granted by the Institutional Review Board (IRB).

The sampling frame for the 2022 GDHS is an updated version of the 2021 Population and Housing Census, prepared by the GSS. The sampling procedure was a stratified two-stage cluster sampling, designed to yield representative results at the national level, for urban and rural areas, and for each of the country’s 16 regions for most DHS indicators.^4^ Given the stratified and complex nature of the DHS data collection processes, inherent measures were adopted to account for nonproportionality in allocation of the sample to the different regions and the possible differences in response rates. Specifically, sampling weights were calculated based on sampling probabilities separately for each sampling stage and for each cluster.^9^ This was necessary to ensure actual representation of the survey results at the national level. Also, the design weights were adjusted for household nonresponse and individual nonresponse in order to obtain the sampling weights for households and for the participants, respectively.^9,29^ The final sampling weights were thus normalized to ensure that the total number of unweighted cases was equal to the total number of weighted cases at the national level for both household weights and individual weights.^9^ The Stata code ‘svy’ was thus applied in our operationalization of the multivariable regression model to properly account for the complex nature of the survey design. Details on the sample design and frame, as well as pretest of survey can be found elsewhere in already published work.^29,30^ Informed consent of the participants was sorted and obtained before the survey was carried out.^4^ Additional information on methods of data collection and survey questionnaire can be found at: https://dhsprogram.com/pubs/pdf/FR387/FR387.pdf

### Measures

Outcome variable: The respondents were questioned: “Have you ever been tested for HIV?” (0 = No, 1 = Yes). Thus, the outcome variable is inherently binary measure of the 2022 GDHS.

Explanatory variables: Guided by both existing literature and the HBM, theoretically relevant explanatory variables were included for analysis. Thus, the educational attainment of respondents, their ages, marital status, wealth, religious affiliation, ethnicity, number of visits to health facility the last 6 months, knowledge of HIV test kits, Knowledge and attitude towords use of PrEP for HIV prevention, employment status, the type of employment, type of earnings from work, type of place of residence (Urban vs Rural), and the region of residence were accounted for. While acknowledging that some of our explanatory variables may fall under more than one construct of the HBM, we have operationalized them in this study as follows: **Perceived Susceptibility**: Age, marital status, and ethnicity can influence how one perceives themselves to be at risk for HIV. **Perceived Barriers**: Wealth, employment status, and rural residence impact access to healthcare. **Perceived Severity & Perceived Benefits**: attitudes toward PrEP shows how serious individuals view HIV/AIDS. Likewise, knowledge and use of HIV test kits for testing may be informed by one’s belief in the effectiveness and benefits of using HIV test kits. **Cues to Action**: Visits to health facilities, knowledge of HIV test kits, and regional differences may serve as triggers to seek health behavior.

### Analytical approach

Both descriptive and inferential statistical analyses have been employed in this study. Statistical distribution table has been used to present socio-demographic characteristics of the study sample. Also, logistic regression models were used to examine the relationship between each predictor variable and the outcome variable. Bivariate and multivariable logistic regression analyses were conducted to ascertain the factors associated with HIVT in the study context. All statistical data analyses were performed in Stata version 18.

## Results

### Sample characteristics

Results for the sample characteristics are presented in Table 1. Majority (56.80%) of the respondents had secondary education, were married (52.23%), from a Christian religious background (61.73%), did not visit a health facility in the preceding 6 months (80.44%), had neither heard of PrEP for HIV prevention (79.67%) nor HIV test kits (76.75%), and never tested for HIV (75.21%). See Table 1 for details on the sample characteristics.

**Table 1.** Descriptive statistics of study sample.

Variable	Frequency distributions (%)
Ever been tested for HIV	
No	5298 (75.21)
Yes	1746 (24.79)
Education	
No education	1186 (16.84)
Primary	923 (13.10)
Secondary	4001 (56.80)
Higher	934 (13.26)
Age	Mean (31.8), SD (12.2), min (15), max (49)
Wealth	
Poorest	1849 (26.25)
Poorer	1553 (22.05)
Middle	1318 (18.71)
Richer	1235 (17.53)
Richest	1089 (15.46)
Marital status	
Never in a union	3047 (43.26)
married/living with partner	3679 (52.23)
Widowed/divorced/separated	318 (4.51)
Religion	
Christianity	4348 (61.73)
Islam	1993 (28.29)
Traditionalist	348 (4.94)
No religion/other	355 (5.04)
Ethnicity	
Akan	2412 (34.24)
Ga/dangme	334 (4.74)
Ewe	765 (10.86)
Guan	369 (5.24)
Mole-dagbani	1811 (25.71)
Grusi	352 (5.00)
Gurma	667 (9.47)
Mande	236 (3.35)
Other	98 (1.39)
Type of employment	
All year	4071 (65.82)
Seasonal	1656 (26.77)
Occasional	458 (7.41)
Currently working	
No	1046 (14.85)
Yes	5998 (85.15)
Type of earnings	
not paid	885 (14.31)
Cash only	4103 (66.34)
Cash and in-kind	992 (16.04)
In-kind	205 (3.31)
Number of visits to health facility last 6 months	
None	5666 (80.44)
Once	838 (11.90)
Twice or more	540 (7.67)
Knowledge and attitude to use PrEP for preventing HIV	
Haven’t heard	5380 (79.67)
Heard but not approve/not sure	414 (6.13)
Heard and approve to take it	959 (14.20)
Knowledge of HIV test kits	
Never heard of HIV test kits	5183 (76.75)
Ever heard of/used HIV test kits	1570 (23.25)
Region	
Western	382 (5.42)
Central	442 (6.27)
Greater accra	493 (7.00)
Volta	335 (4.76)
Eastern	389 (5.52)
Ashanti	491 (6.97)
western North	402 (5.71)
Ahafo	422 (5.99)
Bono	351 (4.98)
Bono east	500 (7.10)
Oti	467 (6.63)
Northern	531 (7.54)
Savannah	548 (7.78)
North East	416 (5.91)
Upper East	464 (6.59)
Upper West	411 (5.83)
Type of place of residence	
Urban	3251 (46.15)
Rural	3793 (53.85)

**Min** = minimum, **Ma**x = maximum, **SD** = standard deviation.

### Bivariate analyses

Educational attainment, age, and wealth were significantly, and positively associated with HIVT (see Table 2). Working men (OR = 2.991; *p* < .001), those paid in cash only (OR = 5.911; *p* < .001) as well as those paid in both cash and kind (OR = 3.524; *p* < .001) reported higher odds of testing compared to the unemployed and the unpaid workers, respectively. Those who visited a health facility at least once in the past 6 months (OR = 2.121; *p* < .001), had knowledge of HIV test kits (OR = 2.995; *p* < .001), aware and approve to take PrEP (OR = 1.762; *p* < .001) were all significantly more associated with testing for HIV compared to those who had not visited health facility in the preceding 6 months, had no knowledge of test kits, and unaware of PrEP, respectively. Moreover, marital status, religion, ethnicity, region, and the type of place of residence (rural vs urban) significantly predicted HIVT at varied levels (see Table 2).

**Table 2.** Bivariate results estimating the uptake of HIV testing among men in Ghana.

Variable	OR (SE)	CI (95%)
**Education** (Ref: No education)		
Primary	1.524 (0.206)**	1.169 1.987
Secondary	2.716 (0.283)***	2.214 3.333
Higher	14.969 (1.773)***	11.868 18.881
**Age**	1.043 (0.002)***	1.038 1.048
**Wealth (**Ref**:** Poorest)		
Poorer	1.820 (0.177)***	1.503 2.204
Middle	2.219 (0.218)***	1.828 2.692
Richer	3.787 (0.359)***	3.144 4.563
Richest	7.692 (0.730)***	6.385 9.266
**Marital status** (Ref**:** Never in a union)		
married/living with partner	2.712 (0.167)***	2.403 3.060
Widowed/divorced/separated	2.185 (0.294)***	1.678 2.846
**Religion** (Ref**:** Christianity)		
Islam	0.587 (0.038)***	0.515 0.668
Traditionalist	0.684 (0.092)**	0.525 0.891
No religion/other	0.371 (0.059)***	0.270 0.509
**Ethnicity** (Ref**:** Akan)		
Ga/Dangme	1.010 (0.130)	0.783 1.302
Ewe	1.006 (0.092)	0.840 1.205
Guan	0.957 (0.119)	0.749 1.224
Mole-dagbani	0.657 (0.048)***	0.568 0.759
Grusi	0.980 (0.124)	0.764 1.258
Gurma	0.501 (0.057)***	0.401 0.627
Mande	0.683 (0.113)*	0.493 0.945
Other	0.735 (0.181)	0.454 1.192
**Currently working** (Ref**:** No)		
Yes	2.991 (0.307)***	2.446 3.658
**Type of employment** (Ref**:** All year)		
Seasonal	0.478 (0.034)***	0.415 0.550
Occasional	0.428 (0.055)	0.332 0.552
**Type of earnings (**Ref: Not paid)		
Cash only	5.911 (0.771)***	4.577 7.634
Cash and in-kind	3.524 (0.518)***	2.641 4.703
In-kind only	1.444 (0.373)	0.870 2.397
**Number of visits to health facility last 6 months** (Ref**:** None)		
Once	2.121 (0.166)***	1.819 2.473
Twice or more	1.931 (0.184)***	1.601 2.330
**Knowledge and attitude to use PrEP for preventing HIV** (Ref**:** Haven’t heard)		
Heard but not approve/not sure to approve	1.734 (0.187)***	1.403 2.144
Heard and approve to take it	1.762 (0.131)***	1.521 2.041
**Knowledge of HIV test kits** (Ref**:** Never heard of HIV test kits)		
Ever heard of HIV test kits	2.995 (0.184)***	2.655 3.379
**Region** (Ref: Western)		
Central	0.661 (0.105)*	0.483 0.904
Greater accra	1.335 (0.194)*	1.003 1.776
Volta	1.089 (0.176)	0.793 1.496
Eastern	1.023 (0.160)	0.752 1.391
Ashanti	0.818 (0.124)	0.608 1.102
western North	0.768 (0.123)	0.561 1.052
Ahafo	0.890 (0.138)	0.656 1.208
Bono	0.776 (0.129)	0.560 1.075
Bono east	0.639 (0.099)**	0.471 0.867
Oti	0.698 (0.109)*	0.513 0.949
Northern	0.417 (0.068)***	0.302 0.576
Savannah	0.402 (0.066)***	0.291 0.555
North East	0.376 (0.067)***	0.264 0.535
Upper East	1.131 (0.168)	0.844 1.514
Upper West	0.806 (0.127)	0.591 1.101
**Type of place of residence** (Ref**:** Urban)		
Rural	0.495 (0.027)***	0.443 0.553

CI = Confidence interval, OR = Odds Ratio, SE = Standard Error,*p < .05,**p < .01,***p < .001.

### Multivariable analyses

Consistent with the results at the bivariate level of analysis, educational attainment, age, and wealth were significantly, and positively associated with HIVT (see Table 3). Compared to those who have never been in a union, married men (OR = 1.723; *p* < .001) reported higher odds of testing. The type of earning was also significantly associated with HIVT. For instance, compared to unpaid workers, those paid in cash only (OR = 2.021; *p* < .001) as well as those paid in both cash and kind (OR = 1.823; *p* < .001) reported higher odds of testing. Also, those who visited a health facility at least once in the past 6 months (OR = 1.615; *p* < .001), had knowledge of HIV test kits (OR = 1.708; *p* < .001), aware and approve to take PrEP (OR = 1.1280; *p* < .001), were all significantly more associated with testing compared to those who had not visited health facility in the preceding 6 months, had no knowledge of test kits, and unaware of PrEP, respectively. Moreover, religion, ethnicity, and the region of residence significantly predicted HIVT at varied levels (see Table 3).

**Table 3.** Multivariable results estimating the uptake of HIV testing among men in Ghana.

Variable	OR (SE)	CI (95%)
**Education** (Ref: No education)		
Primary	1.714 (0.254)***	1.281 2.293
Secondary	2.459 (0.307)***	1.925 3.142
Higher	7.021 (01.057)***	5.227 9.432
**Age (Ref: Continuous)**	1.024 (0.004)***	
**Wealth (**Ref**:** Poorest)		
Poorer	1.518 (0.173)***	1.214 1.900
Middle	1.458 (0.189)**	1.130 1.880
Richer	2.215 (0.306)***	1.688 2.905
Richest	3.371 (0.518)***	2.493 4.558
**Marital status** (Ref**:** Never in a union)		
married/living with partner	1.723 (0.165)***	1.427 2.080
Widowed/divorced/separated	1.311 (0.221)	0.941 1.826
**Religion** (Ref**:** Christianity)		
Islam	0.693 (0.071)***	0.565 0.849
Traditionalist	1.097 (0.183)	0.790 1.523
No religion/other	0.561 (0.100)***	0.395 0.798
**Ethnicity** (Ref**:** Akan)		
Ga/Dangme	0.936 (0.159)	0.670 1.308
Ewe	1.022 (0.140)	0.780 1.338
Guan	1.326 (0.229)	0.944 1.863
Mole-dagbani	1.088 (0.140)	0.846 1.401
Grusi	1.462 (0.260)*	1.031 2.073
Gurma	1.338 (0.217)	0.973 1.840
Mande	1.526 (0.347)	0.976 2.386
Other	1.177 (0.366)	0.639 2.168
**Type of employment** (Ref**:** All year)		
Seasonal	0.909 (0.081)	0.762 1.083
Occasional	0.799 (0.122)	0.592 1.078
**Currently working** (Ref**:** No)		
Yes	1.057 (0223)	0.698 1.600
**Type of earnings (**Ref: Not paid)		
Cash only	2.021 (0.304)***	1.505 2.715
Cash and in-kind	1.823 (0.307)***	1.309 2.538
In-kind only	1.034 (0.291)	0.595 1.798
**Number of visits to health facility in the past 6 months** (Ref**:** None)		
Once	1.615 (0.154)***	1.338 1.949
Twice or more	1.498 (0.176)***	1.1893 1.888
**Knowledge and attitude to use PrEP for preventing HIV** (Ref**:** Haven’t heard)		
Heard but not approve/not sure	1.133 (0.149)	0.875 1.466
Heard and approve to take it	1.1280 (0.104)***	0.940 1.353
**Knowledge of HIV test kits** (Ref**:** Never heard of HIV test kits)		
Ever heard of HIV test kits	1.708 (0.134)***	1.464 1.993
**Region** (Ref: Western)		
Central	0.810 (0.152)	0.560 1.173
Greater accra	0.870 (0.160)	0.606 1.249
Volta	1.071 (0.239)	0.690 1.661
Eastern	1.035 (0.199)	0.709 1.511
Ashanti	0.843 (0.150)	0.594 1.196
western North	0.937 (0.179)	0.644 1.364
Ahafo	1.220 (0.230)	0.843 1.766
Bono	0.907 (0.184)	0.609 1.352
Bono east	0.964 (0.187)	0.658 1.411
Oti	0.938 (0.194)	0.624 1.409
Northern	0.726 (0.162)	0.468 1.125
Savannah	1.012 (0.225)	0.653 1.567
North East	0.877 (0.215)	0.542 1.419
Upper East	1.818 (0.383)**	1.202 2.749
Upper West	1.489 (0.326)	0.970 2.287
**Type of place of residence** (Ref**:** Urban)		
Rural	0.931 (0.077)	0.791 1.096

CI = Confidence interval, OR = Odds Ratio, SE = Standard Error,*p < .05,**p < .01,***p < .001.

## Discussion

Guided by the Health Belief Model, this study explored the health behaviour of men (aged 15–49 years) towards HIVT in Ghana. The findings present valuable insights for the design of targeted interventions that will improve testing among men. At the bivariate level of analysis, the results indicates that 75.21% (representing about 5298 males out of the 7044 respondents) never tested for HIV. This finding presents a worrying concern given the enormous efforts by the government and Ghana AIDS Commission to normalize HIVT among the general population. Such poor testing rates have adverse implications for achieving the UNAIDS set target of 95-95-95 by 2030 in Ghana. This signals a wake-up call to intensify and reorient existing HIV campaigns and awareness creation that will ensure that more men take up HIVT. In the subsequent paragraphs, we discuss our key findings within the HBM theoretical framework.

Consistent with prior studies in Ghana**,** men with higher educational attainment, older age, and better socioeconomic status (SES),^20^ had higher odds of HIVT than their counterparts. While higher educational attainment and better economic status are not synonymous to perceived susceptibility, available studies suggest that these factors can work to increase an individual’s perceived risk of diseases like HIV infection.^31,32^ The higher likelihood of testing among men with better SES further support the notion that wealth is a major determinant of health, especially in developing and resource-constrained countries. A prior study in Ghana suggest that socioeconomic status tend to shape different levels of vulnerabilities and privileges in society, particularly, health information access,^15^ consequently influencing health behaviours. Moreover, men with low SES who may be reluctant to utilize public testing services for reasons pertaining to privacy and the stigma associated with such testing services, may also lack the financial capacity to afford test kits for self-testing. Thus, notwithstanding the fact that Ghana has made a slow but steady progress towards universal testing, SES still plays a crucial role in HIVT disparities. Furthermore, the finding that the likelihood of ever testing for HIV increases with age aligns with the findings of a recent study on the trends of HIVT and knowledge of status across SSA countries.^6^ The total number of lifetime sexual partners, or the tendency of being in multiple sexual relationships is shown to be high among older adults,^33^ which may lead to a high perceived risk of infection, and the need to test and know one’s status. These findings underscore the need to target and identify young, uneducated men, including those with low SES, with HIVT programs.

The findings also indicate that married men and those who earned income (either in cash or both cash and kind) were associated with higher odds of HIVT. Individuals in stable relationships may recognize the benefits of HIVT for the well-being of their families, while those with stable incomes may overcome perceive financial barrier to testing. Studies have indicated that the desire to get married is a major factor that prompt people to get tested in the Ghanaian context.^34^ Tenkorang et al.^35^ in Ghana observed that some religious denominations demands mandatory HIVT by couples as part of the sanctioned pre-wedding activities. These dynamics may be working to expose more married men to HIVT relative to their unmarried counterparts.

Closely related, the findings indicate that religion and ethnicity were associated with the uptake of HIVT among men in Ghana. Informed by context, some sociocultural factors can either serve as facilitators, or potential barriers, influencing individuals’ decisions to undergo testing underpinned by community norms, cultural beliefs, and regional healthcare accessibility. Our findings demonstrated that respondents without any religious affiliation, and those form Islamic religious background were less likely to undergo HIVT compared to their Christian counterparts. This finding may be explained by earlier studies in Ghana suggesting that some Christian denominations serve as vehicles for the dissemination of HIV information, including issues related to HIV/AIDS prevention.^35^ Specifically on HIVT, prior studies have also highlighted the crucial role of some religious denominations in the fight against HIV through mandatory HIVT imposed on couples who intend getting married, and organize periodic HIVT for their entire congregation.^36^ Thus, while the strengths of major religious institutions can be harnessed for the promotion of testing among congregants, people without religious affiliation must not be left out in the design of testing initiatives. Also, Islamic religious institutions and organization must be encouraged to engage Muslims on topics of sexual health, such as HIV prevention through testing. Furthermore, the findings suggest that the participants from the Grusi ethnic background were more likely to have ever tested for HIV than those from the Akan ethnic background. Prior studies have also highlighted ethnic disparities in HIVT in Ghana.^37^ Men are in the Ghanaian context are notably the preservers of ethno-cultural beliefs in patriarchal societies; hence the tendency of most men being influenced by ethnic and cultural beliefs that are apar from modern preventive healthcare such as HIVT cannot be underestimated. Thus, ethnic beliefs that discourages HIVT, and consequently amounting to ethnic disparities in testing uptake, must be identified and addressed as parts of efforts to scale up testing among men.

‘Cue to Action,’ we observed that individuals who recently visited a health facility, had knowledge of HIV test kits, and are aware and approved of Pre-Exposure Prophylaxis (PrEP), significantly reported higher odds of testing. This finding not only reaffirms the HBM assessment that information is required to trigger engagement in healthy actions. Cues to action can take various forms, including media messages, individual experiences, advice from healthcare professionals, or observations, among others. In this study, men who visited healthcare facilities in the past 6 months reported higher odds of HIVT, highlighting the need to encourage routine visits to healthcare facilities among men. Contact with health professionals is an important source of health information especially among people without the ability to read and comprehend health information on their own. Through such contacts, people may be informed about preventive measures (including PrEP, use of HIV test kits) and may view testing as a proactive health behavior. Although health facility visits and establishment of contact with health professionals exposes people to important health information, in Ghana, factors such as perceptions of quality of care and HIVT, as well as privacy and confidentiality in healthcare professionals, are potential barriers to accessing testing services.^38^ The creation of awareness about HIV test kits, and promotion of self-testing is an important alternative. Though the results of most self-tests stand lower chances of being disclosed due to fear of stigmatization (especially when positive), self-testing is a highly recommended measure for many to know their status and either take action to reduce the risk of sexual infection or seek treatment when positive.

Regarding the region of residence and odds of HIVT, the findings indicates that residents of the Upper East region were more likely to ever been tested for HIV relative to those from the Western Region. A study in the Upper East region have demonstrated that there is a high level of men involvement in antenatal care during their partners’ pregnancy.^39^ Given that the mandatory testing policy for pregnant women also encourages the testing of their partners,^8^ men who partake in antenatal care would most likely be tested as well. Just like there is a policy to ensure mandatory testing of pregnant women as part of efforts to prevent mother-to-child transmission, there should be deliberate health policies that create equal opportunities for men to get tested for HIV. Overall, the Estimated total number of People Living with HIV (PLHIV) by Region (All Ages) is lower in the Upper East region (5776) relative to the Western region (19,731), Central (22,021), Eastern (42,446), Ashanti (76,672), and the Greater Accra region (94,104).^7^ Thus, these regional differences in HIV prevalence rates or incidences, and testing uptake must be considered and integrated into national efforts towards universal testing.

## Conclusion

The findings emphasize the need for policies and interventions that enhance the perceived benefits of testing among men, reduce perceived barriers, and utilize appropriate cues to action (e.g., encouraging regular visits to health facilities) within the diverse sociodemographic contexts of Ghana. Ensuring active engagement of men in HIV prevention efforts would not only benefit men, but also, their sexual partners through reduction in sexual infection of these partners, and by extension, reducing mother-to-child transmission.
